# Ti/TiO_2_/SiO_2_ multilayer thin films with enhanced spectral selectivity for optical narrow bandpass filters

**DOI:** 10.1038/s41598-021-03935-z

**Published:** 2022-01-07

**Authors:** Dongju Kim, Kang Min Kim, Hyuksu Han, Junho Lee, Deahyeon Ko, Kyoung Ryeol Park, Kyu-bong Jang, Dongwon Kim, Jennifer Sue Forrester, Seung Hwan Lee, Jong Cheol Kim, Sungwook Mhin

**Affiliations:** 1 Department of Advanced Materials Engineering, Kyonggi University, Suwon, 16227 Republic of Korea; 2 Korea Institute of Industrial Technology, 137-41 Gwahakdanji-ro, Gangneung-si, Gangwon 25440 Republic of Korea; 3 Department of Energy Engineering, Konkuk University, 120 Neungdong-ro, Gwangjin-gu, Seoul, 05029 Republic of Korea; 4 Green Materials & Process R&D Group, Korea Institute of Industrial Technology, 55 Jongga-ro, Jung-gu, Ulsan, 44413 Republic of Korea; 5 School of Materials Science and Engineering, Inha University, 25 Younghyun-Dong, Incheon, 405-751 Republic of Korea; 6 Analytical Instrument Facility, North Carolina State University, Raleigh, NC 27695 USA; 7 Department of Mechanical Engineering, Hanyang University, 222, Wangsimni-ro Seongdong-gu, Seoul, 04763 Republic of Korea; 8 Daegu Mechatronics & Materials Institute, 11 Seongseogongdan-ro, Daegu, 42714 Republic of Korea

**Keywords:** Engineering, Materials science, Nanoscience and technology, Optics and photonics

## Abstract

Thin film-based optical sensors have been attracting increasing interest for use in developing technologies such as biometrics. Multilayered dielectric thin films with different refractive indices have been utilized to modulate the optical properties in specific wavelength bands for spectral selectivity of Thin Film Narrow Bandpass Filters (TFNBFs). Progress in TFNBF design has been made with the incorporation of metallic thin films. Narrower bandwidths with higher transmittance have been achieved in specific spectral bands. In this work, Ti/TiO_2_/SiO_2_ based multilayer thin films were prepared using pulsed-DC reactive sputtering. Computer simulations using the Essential Macleod Program allowed the optimal number of layers and thickness of the multilayer thin films to be determined to efficiently tailor the optical path transmitting specific wavelength bands. The addition of Ti metal layers within dielectric (TiO_2_/SiO_2_) multilayer thin films significantly changes the cutoff frequency of transmittance at specific wavelengths. Representative 26 multilayer films consisting of Ti, TiO_2_, and SiO_2_ show lower transmittance of 10.29% at 400 nm and 10.48% at 680 nm. High transmittance of 80.42% at 485 nm was observed, which is expected to improve the spectral selectivity of the TFNBF. This work provides a contribution to future simulation based design strategy based on experimental thin film engineering for potential industrial development opportunities such as optical biometrics.

## Introduction

Biometrics is an expanding technology used to measure unique identity verification characteristics in humans including fingerprints, facial and iris features^1^. For optical biometrics, a light is emitted on a face or fingerprints, and is reflected back to the bandpass filter eliminating ambient light, which transmits the light with high signal-to-noise ratio in the desired spectral band to a detector. Notably, a narrow bandpass filter is defined as an optical filter that passes one or more desired wavelength bands while blocking others. Thin film narrow bandpass filters (TFNBFs) are an essential component for the control of the recognition rate for biometrics, which can manipulate the specific transmittance for contrast tuning of the image^2,3^. TFNBFs commonly consist of multiple layered thin films with different refractive indices, which produce differences in the spatial and spectral distribution of light induced by the thin-film interference effect^4^.

Interference of light can be determined by differences in the refractive index between alternating layers of TFNBFs. Large differences in the refractive index between constituent layers is preferred for contrast enhancement of an image. The TiO_2_/SiO_2_ thin film system is considered an excellent candidate for TFNBFs due to the large differences in refractive indices (~ ∆0.95) in a wide range of wavelengths (250 nm to 3000 nm)^5,6^. Stoichiometric and microstructural engineering of the TiO_2_ and SiO_2_ layers further increases differences in the refractive index, which may alter transmittance at specific wavelengths^5–8^. Investigations of different aspects of the TiO_2_/SiO_2_ system, including microstructure, crystallography, chemical composition and processing are important to determine how the refractive index can be modified^9–12^.

Within the TiO_2_/SiO_2_ dielectric system, the introduction of a metal layer can modify the optical properties of transmitted light in the TFNBF due to surface plasmon resonance between dielectric and metal thin films. Surface plasmon polaritons excited by light propagates along the metal surface and decay exponentially at the interface between metal and dielectric thin films, which can lead to transmittance loss of the TFNBF at specific wavelengths^13^. A Ti metal layer can exhibit a high refractive index (~ 1.98) and extinction coefficient (~ 3.05) in a range of wavelengths between 300 and 2000 nm^14–16^. For example, the introduction of a metal layer within dielectric layers results in unique optical properties including high visible transmission, near-infrared heat shielding, and reflective filtering^17,18^.

Multilayered thin films can be prepared using processing techniques such as chemical vapor deposition (CVD), evaporation, and sputter deposition. Difficulties can arise with the final processed materials when using CVD and evaporation. Columnar structures with micro-pores can develop via capillary action due to moisture absorption when the films are exposed to the ambient atmosphere after processing is completed, which can induce unintended transmittance or reflectance when light travels across multilayers^19,20^. By comparison, sputter deposition can provide dense microstructures due to the higher energy of adatoms for boosting surface diffusion on the substrate^21^. In addition, the implementation of high-density plasma (HDP) and a cylindrical design of the target for sputter deposition can further manipulate the optical constants (*n*, *k*) and chemical composition of thin films^22,23^.

Herein, Ti/TiO_2_/SiO_2_ multilayered thin films prepared using HDP pulsed-DC reactive sputter deposition are presented, and their suitability for application as thin film narrow bandpass filters is discussed. With the application of HDP, the stoichiometry of the TiO_2_/SiO_2_ dielectric films was precisely controlled. The use of a pulsed-DC power supply provides a smooth surface and dense microstructure of the Ti/TiO_2_/SiO_2_ multilayers. The effect that the sputtering power has on the crystal structure and refractive index of the deposited films is presented. The cutoff frequency of the Ti/TiO_2_/SiO_2_ multilayer is investigated from 300 to 1100 nm, which is within the range of potential application as TFNBFs.

## Results and discussion

**Figure 1:**
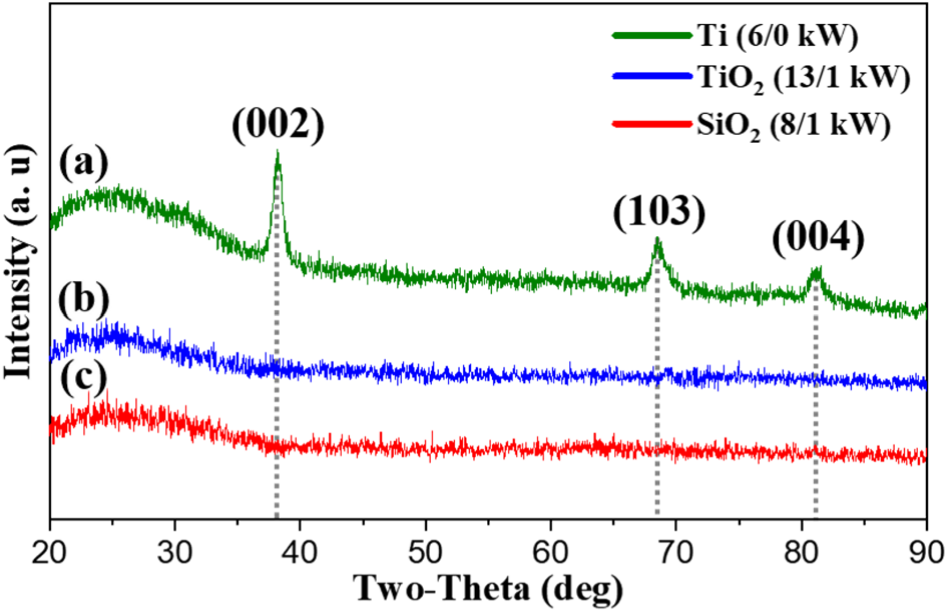
XRD patterns of (**a**) Ti thin film (crystalline), (**b**) TiO_2_ thin film (amorphous), and (**c**) SiO_2_ thin film (amorphous).

XRD patterns of the Ti, TiO_2_, and SiO_2_ thin films (with different sputtering powers) are shown in Fig. 1. Both TiO_2_ and SiO_2_ thin films show predominantly an amorphous phase, while the Ti metal thin films show crystalline phases. An amorphous phase was observed in the TiO_2_ and SiO_2_ thin films regardless of sputtering power, as shown in Fig. S1. Pulsed-DC and RF sputtering power are abbreviated as X/Y kW. When the Ti thin film was deposited using a sputtering power of 6/0 kW, a reflection is present at 81.07° 2θ coinciding with the absence of the (110) reflection (62.79°). The reflection at 81.07° 2θ can be identified as the (004) plane of the β-Ti (bcc) structure. This suggests that there is preferential film growth along the *c*-axis, which may be due to a lower surface energy of the (001) than other lattice planes^24–26^.

**Figure 2:**
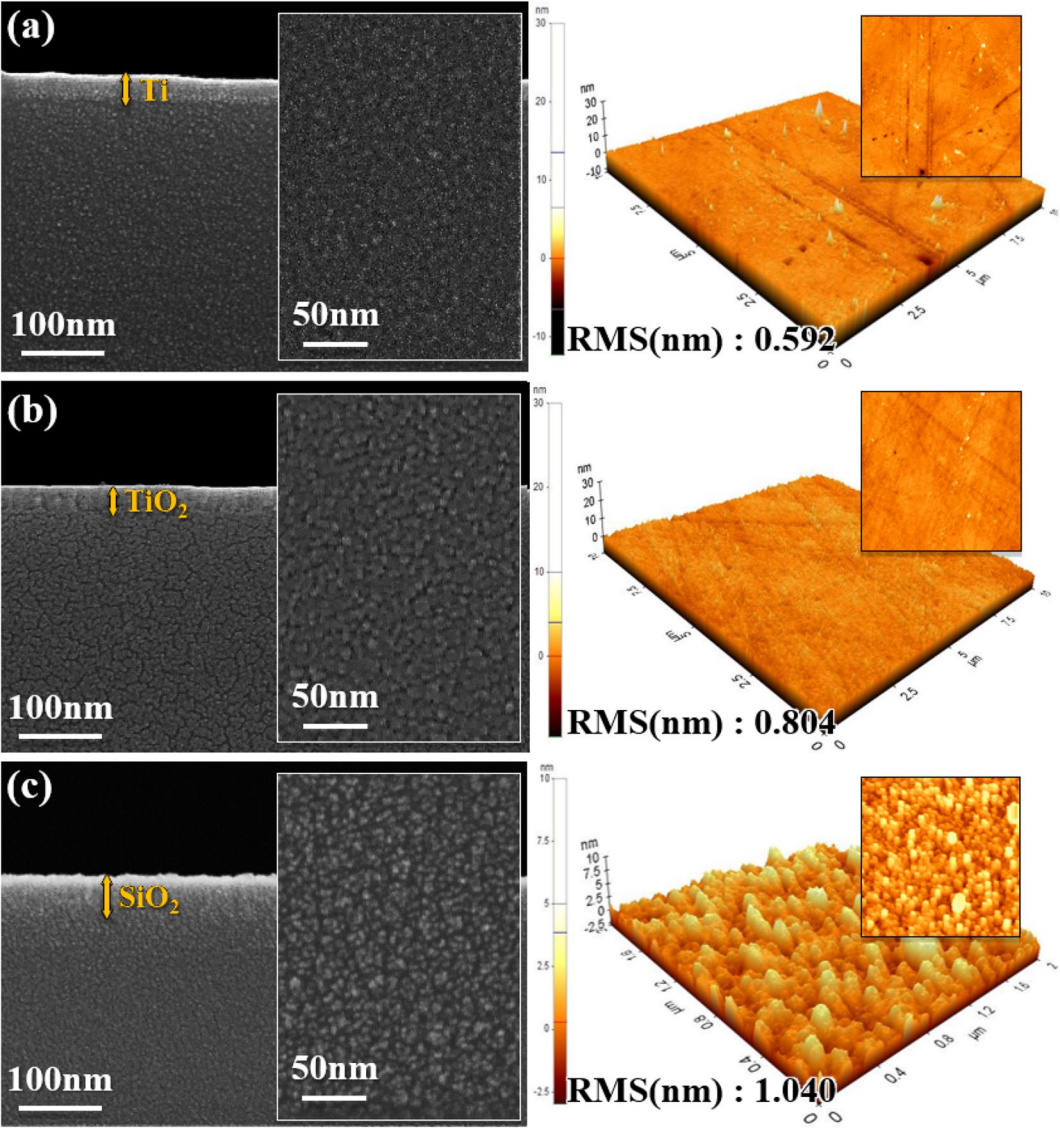
SEM secondary electron micrographs and AFM images of (**a**) Ti thin film, (**b**) TiO_2_ thin film, and (**c**) SiO_2_ thin film.

Cross-sectional and surface micrographs obtained from AFM and SEM show that high sputtering power increases roughness and also a more dense film structure. Figure 2a–c show the cross-sections of optimized Ti, TiO_2_, and SiO_2_ thin films prepared using sputtering power 13/1 kW, 8/1 kW and 6/0 kW, respectively. All thin films show smooth surfaces, confirmed by low RMS obtained from AFM measurements. Additional cross-sectional images of the Ti, TiO_2_, and SiO_2_ thin films are provided in Fig. S2. It is well known that pulsed-DC supply is advantageous for obtaining dense and uniform structures, because surface diffusion of the sputtered particles on the substrate promotes homogenous film growth^27,28^. Accordingly, the low roughness and dense structure of the TiO_2_, SiO_2_ and Ti thin films presented here suggests that Pulsed-DC sputtering is beneficial for preparation of smooth and dense thin films.

**Figure 3:**
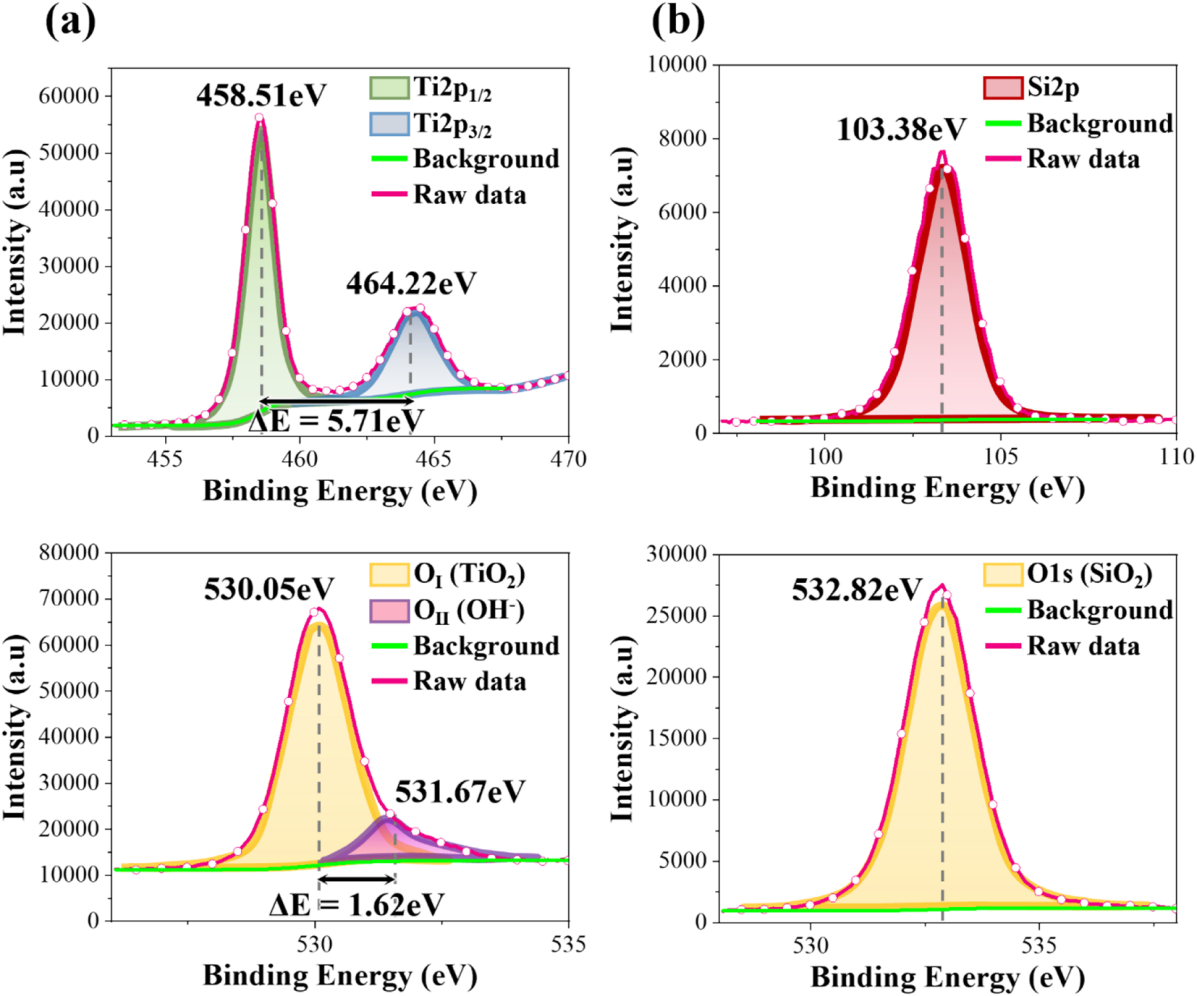
XPS spectra and atomic concentrations of (**a**) TiO_2_ thin film, and (**b**) SiO_2_ thin film.

Elemental composition and chemical states of the constitutive elements are important factors in the resultant microstructure, refractive index and extinction coefficient of thin films. XPS spectra of a TiO_2_ thin film are shown in Fig. 3a. Two major peaks at 458.51 and 464.22 eV can be assigned to Ti 2p_3/2_ and 2p_1/2_ energy levels, respectively. The binding energy of Ti_2p_ indicates that the oxidation state of the Ti is 4+^25,29^. The O1s spectra show two major peaks at 530.05 eV and at 531.67 eV, which can be attributed to O_I_ and O_II_, respectively^30^. The O_I_ Peak corresponds to O^2−^ in the lattice sites of the TiO_2_ structure, while the O_II_ Peak is assigned to OH^−^ bonded to Ti^3+^. A dense microstructure is commonly formed in thin films when the ratio of O_I_ to O_II_ is higher. The ratio of O to Ti in the film is 1.97, which indicates the chemical composition of the thin film is TiO_1.97_. XPS spectra of the SiO_2_ thin film is shown in Fig. 3b. Si2p at 103.38 eV and O1s at 532.82 eV can be assigned to Si^4+^ and O^2−^, respectively^31^. The ratio of O to Si is 1.89, indicating the formation of SiO_1.89_ thin film.

**Figure 4:**
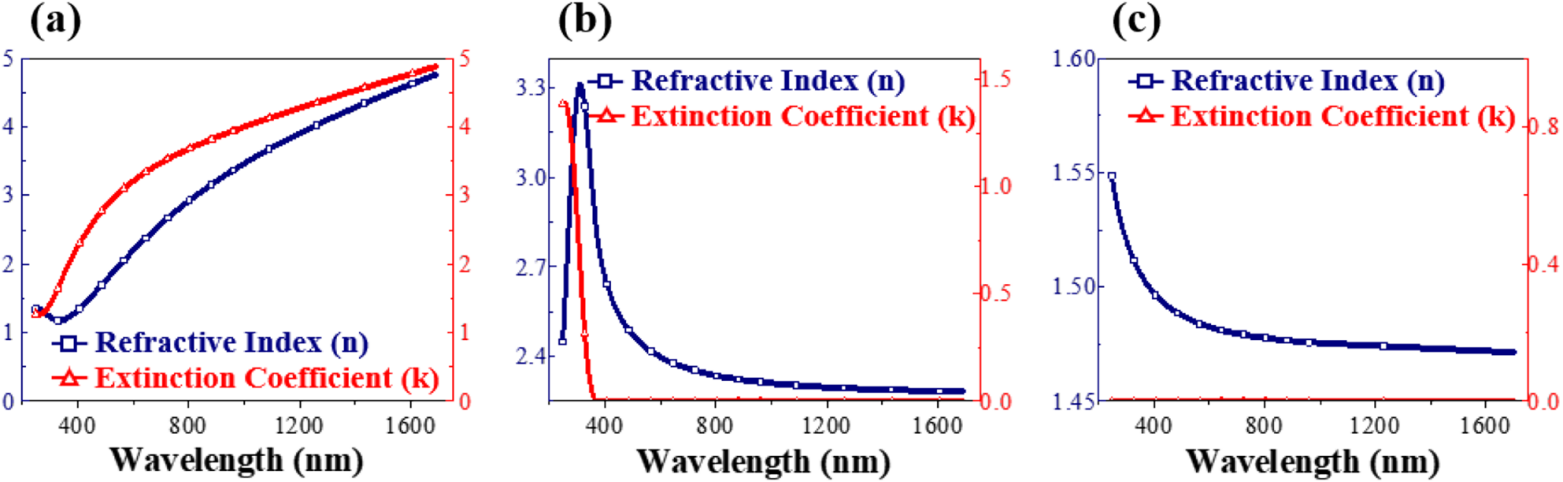
Refractive Index (n) and Extinction Coefficient (k) of (**a**) Ti thin film, (**b**) TiO_2_ thin film, and (**c**) SiO_2_ thin film.

The refractive indices ($n$) and the extinction coefficients ($k$) of Ti, TiO_2_ and SiO_2_ single-layer films were measured in the wavelength range 300 nm to 1800 nm by Ellipsometer, and a selection of the results is shown in Fig. 4 (results at other selected wavelengths are provided in Fig. S3). The refractive index at 550 nm wavelength of Ti is 2.43, TiO_2_ is 1.48, and SiO_2_ is 1.99. The extinction coefficients at 550 nm for TiO_2_, SiO_2_ and Ti are 0.00, 0.00 and 3.05, respectively. The high refractive index and extinction coefficient of the metal film can be attributed to large absorption of the incident radiation through electronic conduction in the metal film^32^. It is suggested that the incorporation of metal films into multilayer dielectric films can efficiently modulate the optical properties, and thus achieve desired optical properties of the narrow bandpass filter.

Transmittance and reflectivity of the multilayer thin films may be estimated using the following relation, which is described by the Fourier Transform relationship^33^:

1 $$\mathrm{ln} \left (\frac{n(x)}{{n}_{0}} \right)=\frac{j}{\pi }\int \frac{\tilde{Q }(\sigma )}{\sigma }{e}^{-j2\pi \sigma x}d\sigma$$

where *n*(*x*) is the refractive index profile, and $\tilde{Q }(\sigma )$ is the complex function of the transmittance or reflectivity. The effective thickness, *x*, can be calculated by:

2 $$x=2{\int }_{0}^{z}n\left(z\right)dz$$

It can be shown from Maxwell’s equations that the transmittance of the thin film based optical filter can be expressed as Eq. (3). $d\tilde{Q }$ is integrand given in Eq. (4).

3 $$\frac{1}{t(\sigma )}={e}^{-j2\pi \sigma x}\{1+{\int }_{0}^{x}d\tilde{Q }\left({x}_{1}, \sigma \right){\int }_{0}^{{x}_{1}}d{\tilde{Q }}^{*}\left({x}_{2}, \sigma \right)+\iiint \cdots +\cdots \}$$

4 $$\tilde{Q }\left(\sigma \right)=\int \frac{1}{2n}\frac{dn}{dx}{e}^{j2\pi \sigma x}dx$$

where the complex function $\tilde{Q }(\sigma )$ is expressed as:

5 $$\tilde{Q }\left(\sigma \right)=Q\left(\sigma \right){e}^{j\varnothing (\sigma )}$$

In TFNBFs, the cutoff frequency of transmittance can be determined by the complex function $\tilde{Q }(\sigma )$ depending on the refractive index *n*(*x*) and effective thickness *x*. Accordingly, the application of a metal film between dielectric films as well as thickness control can tune the transmittance in a specific wavelength, thereby selectively controlling the reflectance or absorption of light in a specific wavelength^34–37^.

**Figure 5:**
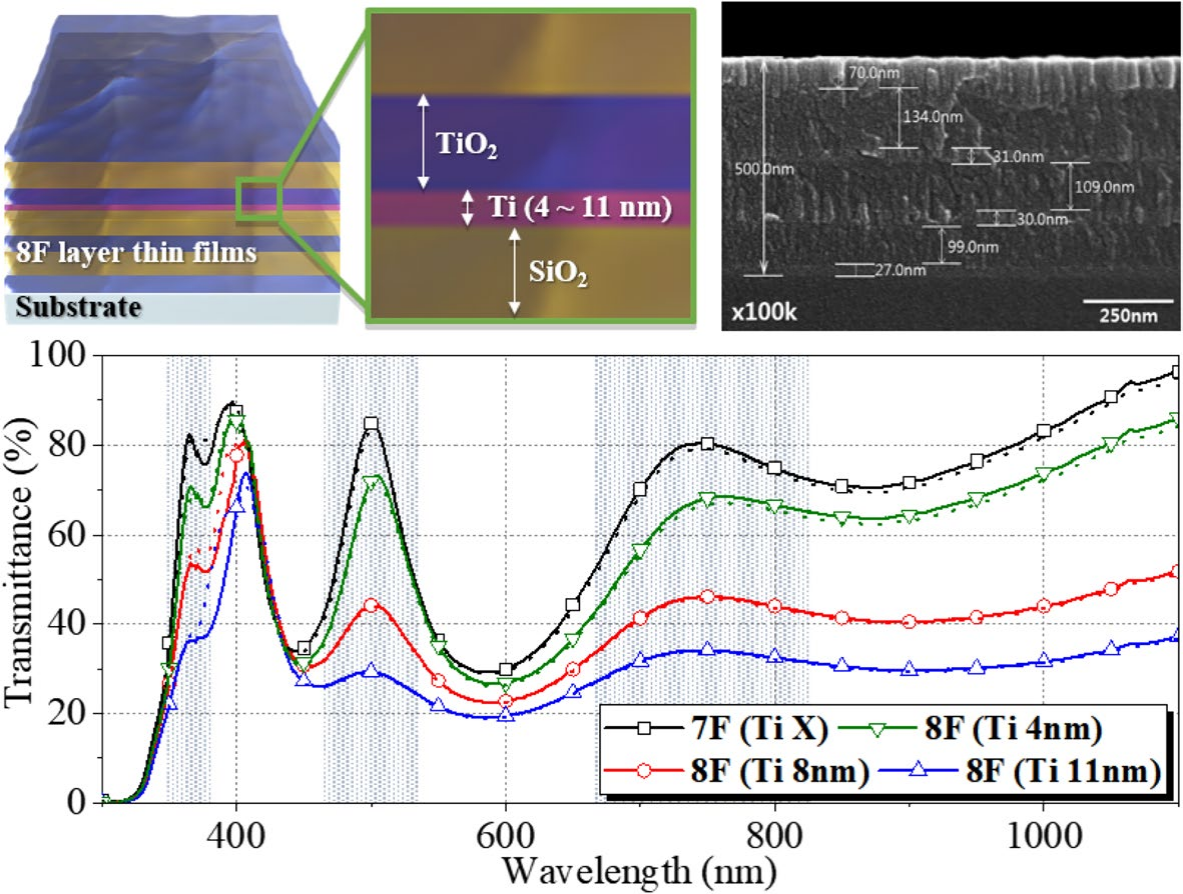
Morphology and transmittance spectra of multi-layered structure of (Ti)/TiO_2_/SiO_2_ films with 7–8 layers. Simulated transmittance spectra are highlighted by the dotted line.

The number of thin films and effective thickness for optimized transmittance of TFNBFs was obtained using the Essential Macleod Program (EMP). We chose 8-layered thin films for computational calculation which demonstrates the effect of a Ti layer on the optical properties of the dielectric (TiO_2_ and SiO_2_) based multilayered thin films. Based on the EMP simulations, the thickness of each Ti, TiO_2_, and SiO_2_ thin film was precisely controlled in the deposition of 8-layered thin films, as shown in Fig. 5. Transmittance of 8-layered thin films was investigated in the wavelength range of 300 nm to 1100 nm. Different thicknesses of the Ti thin film were deposited between 4F SiO_2_ (108.0 nm) and 5F TiO_2_ (20.0 nm) as shown in Figs. 5 and S4. For comparison, transmittance of 7-layered thin films consisting of dielectric (TiO_2_ and SiO_2_) thin films only is also presented. The transmittance in 7-layered thin films was 82.29% (at 360 nm wavelength), 89.72% (at 400 nm), 84.81% (at 500 nm) and 79.44% (above 750 nm). With the addition of Ti metal films with different thicknesses (8-layered thin films), transmittance significantly changed in specific wavelengths as shown in Fig. 5, which shows a transmittance of 8-layered thin films was 33.71% (at 360 nm wavelength), 71.08% (at 400 nm), 29.34% (at 500 nm), and 33.58% (above 750 nm). It appears that thicker Ti metal films result in larger $\Delta T$. Simulated transmittance at specific wavelengths, highlighted by the dotted line, matches well with experimental data, indicating that the inclusion of metallic thin films with different thickness into dielectric thin films can effectively tailor the optical properties of the TFNBF.

**Figure 6:**
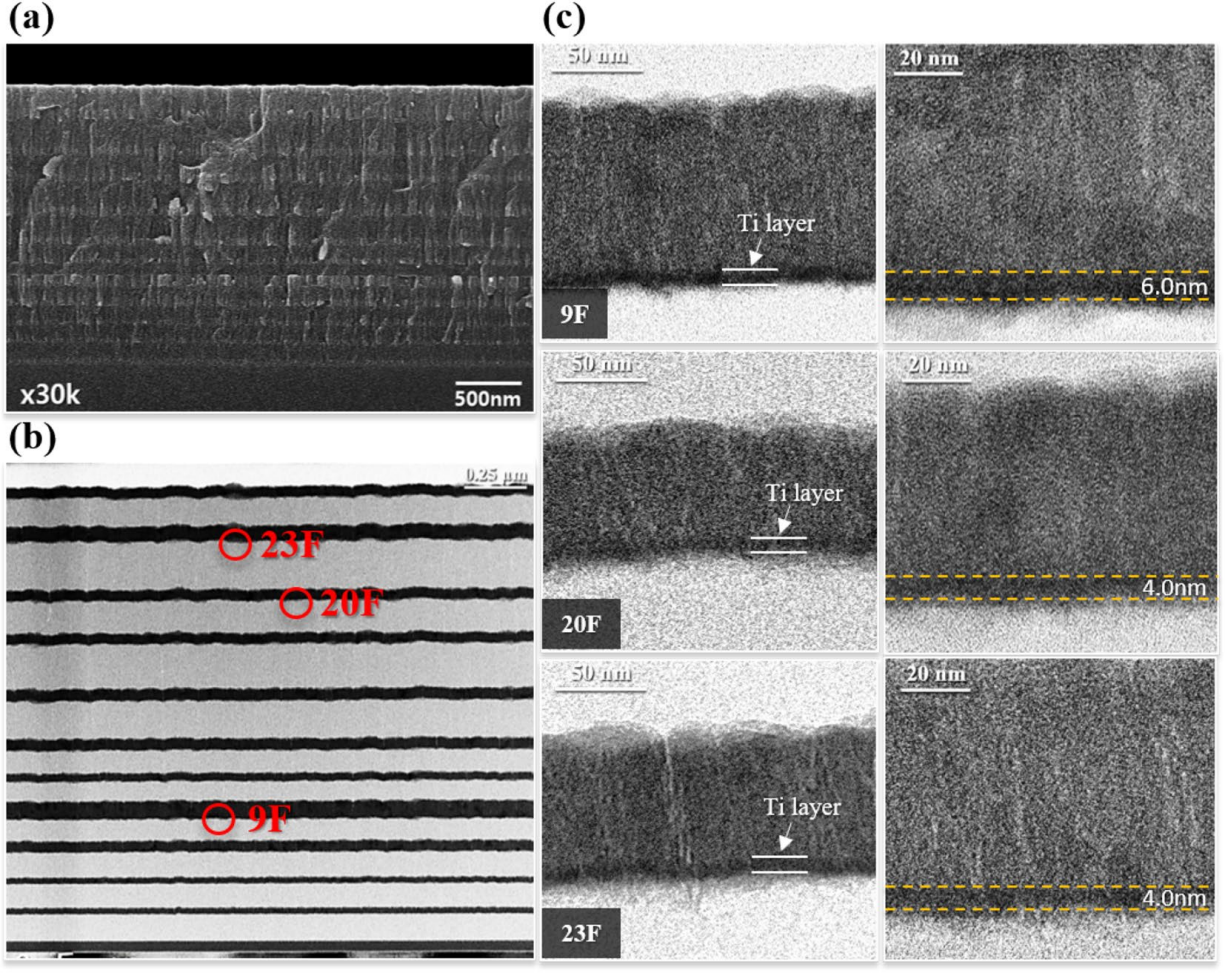
Morphology and layer thickness multi-layered structure of Ti/TiO_2_/SiO_2_ films with 26 layers: (**a**) SEM secondary electron micrograph of a cross-section of a multilayer film, (**b**) Low magnification TEM image of a multilayer film, and (**c**) High magnification TEM micrograph of a multilayer film.

**Figure 7:**
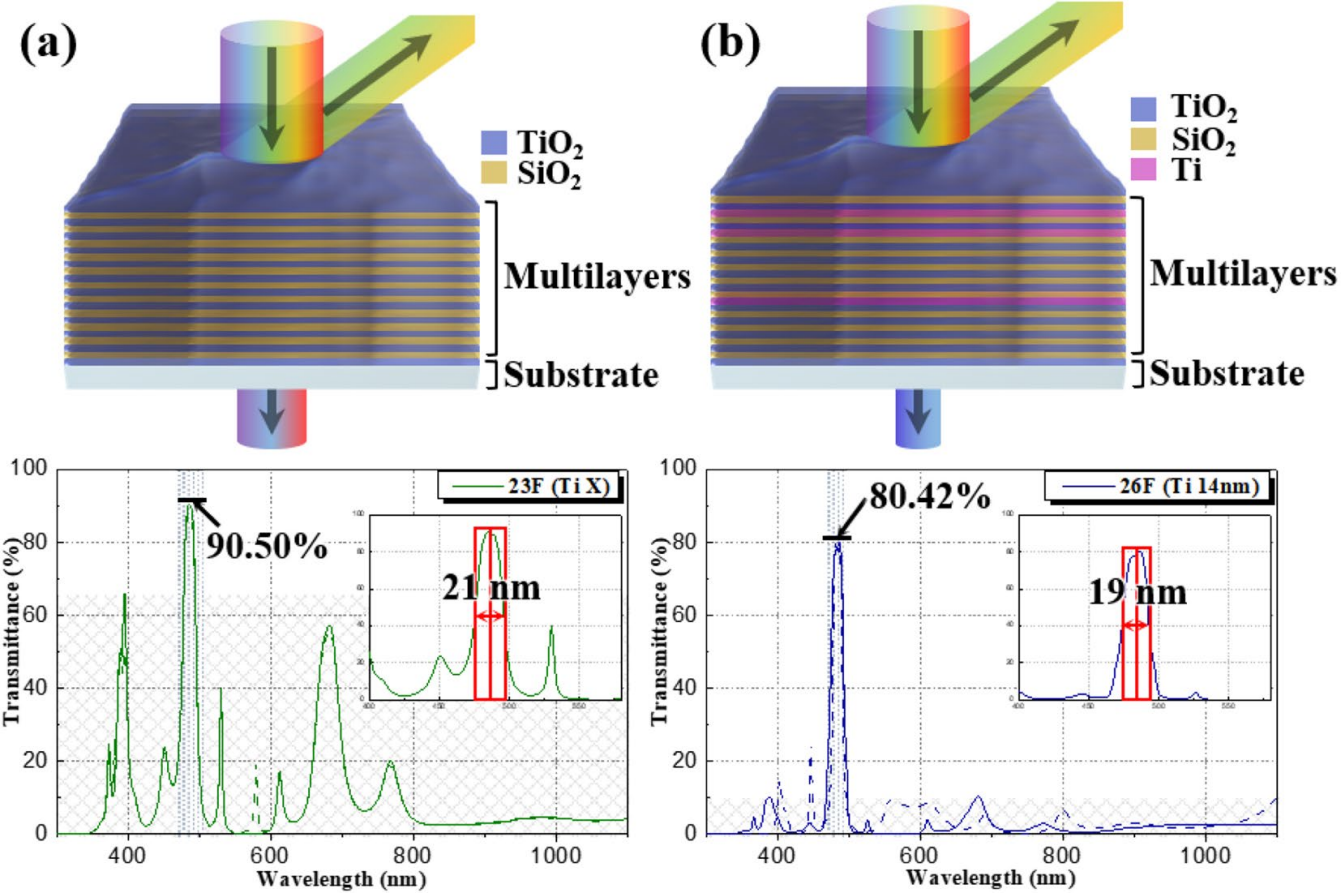
Transmittance spectra of multilayer films of (**a**) TiO_2_/SiO_2_ films with 23-layers, and (**b**) Ti/TiO_2_/SiO_2_ films with 26-layers. Simulated transmittance spectra are highlighted by the dotted line.

**Figure 8:**
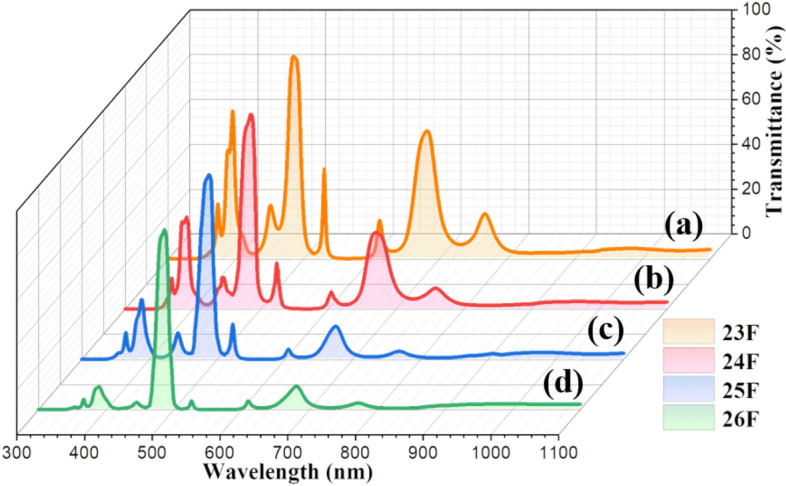
Transmittance spectra of multilayer films of (**a**) TiO_2_/SiO_2_ films with 23-layers, (**b**) Ti/TiO_2_/SiO_2_ films with 24-layers. (**c**) 25-layers, and (**d**) 26-layers.

Based on the EMP simulations, transmittance at 485 nm can be selectively modulated when thickness and a sequence of inserted Ti metal films is precisely controlled in the deposition of 26-layers thin films: the deposition sequence and thickness of the Ti, TiO_2_ and SiO_2_ thin films are shown in Fig. 6 and Table 1. For comparison, 23-layered TiO_2_/SiO_2_ films were also prepared as shown in Fig. S5 and Table 1. There was little difference in the total thickness between 23-layers and 26-layers thin films. Interfacial diffusion among thin films was not observed in the 26-layers thin films. Transmittance of the 23-layer and 26-layer thin films in the wavelength range from 300 and 1100 nm was evaluated, as presented in Fig. 7. For 23-layer films, transmittance of 90.5% with a FWHM of 21 nm was observed at low cutoff frequency (485 nm). Transmittance of 65.98% and 57.21% was observed at high cutoff frequency of 400 nm and 680 nm, respectively. With the insertion of Ti metal layers, a high transmittance of 80.42% with FWHM of 19 nm at 485 nm was observed. A lower transmittance of 10.29% and 10.48% was observed at 400 nm and 680 nm, respectively. That is, increased $\Delta T$ at both a low cutoff frequency (485 nm) and a high cutoff frequency (400 nm, 680 nm) is achieved in the 26-layer thin films. Simulated transmittance spectra of multilayered thin films with different metal layers including Si, Ag, Zn and Al are presented in Fig. S6, and this comparison indicates that Ti is effective as the metallic layer to obtain selective transmittance at specific wavelengths. Experimental results matched well with simulated optical properties as shown in Fig. 7. Transmittance spectra of Ti/TiO_2_/SiO_2_ multilayer thin films with an increasing number of Ti layers is shown in Fig. 8, which implies that Ti layers are beneficial to improve the spectral selectivity. However, as shown in Fig. S7, further increasing the number of Ti layers gradually decreases transmittance in the wavelength range of 300 nm to 1100 nm providing a poor signal-to-noise ratio. For application of multilayer thin films to narrow bandpass filters, a square bandwidth with a steep slope of the transmittance at specific wavelength is essential, highlighted in the red squares in the inset of Fig. 7.

**Table 1:** Simulated layer thickness of TiO_2_/SiO_2_ multilayer films with 23-layers and Ti/TiO_2_/SiO_2_ multilayer films with 26-layers.

23-layers TiO_2_/SiO_2_ thin films							
(6F) SiO_2_	99.8 nm	(12F) SiO_2_	90.6 nm	(18F) SiO_2_	129.0 nm		
(5F) TiO_2_	43.7 nm	(11F) TiO_2_	53.2 nm	(17F) TiO_2_	72.9 nm	(23F) TiO_2_	78.6 nm
(4F) SiO_2_	96.0 nm	(10F) SiO_2_	78.4 nm	(16F) SiO_2_	176.6 nm	(22F) SiO_2_	110.4 nm
(3F) TiO_2_	45.3 nm	(9F) TiO_2_	125.4 nm	(15F) TiO_2_	82.8 nm	(21F) TiO_2_	105.1 nm
(2F) SiO_2_	107.5 nm	(8F) SiO_2_	86.6 nm	(14F) SiO_2_	149.1 nm	(20F) SiO_2_	186.9 nm
(1F) TiO_2_	85.6 nm	(7F) TiO_2_	80.1 nm	(13F) TiO_2_	78.6 nm	(19F) TiO_2_	73.6 nm
Total 2236.0 nm							

26-layers Ti/TiO_2_/SiO_2_ thin films							
(7F) TiO_2_	80.1 nm	(14F) TiO_2_	78.6 nm	(21F) TiO_2_	73.6 nm		
(6F) SiO_2_	99.8 nm	(13F) SiO_2_	90.6 nm	(20F) Ti	4.0 nm		
(5F) TiO_2_	43.7 nm	(12F) TiO_2_	53.2 nm	(19F) SiO_2_	129.0 nm	(26F) TiO_2_	78.6 nm
(4F) SiO_2_	96.0 nm	(11F) SiO_2_	78.4 nm	(18F) TiO_2_	72.9 nm	(25F) SiO_2_	110.4 nm
(3F) TiO_2_	45.3 nm	(10F) TiO_2_	125.4 nm	(17F) SiO_2_	176.6 nm	(24F) TiO_2_	105.1 nm
(2F) SiO_2_	107.5 nm	(9F) Ti	6.0 nm	(16F) TiO_2_	82.8 nm	(23F) Ti	4.0 nm
(1F) TiO_2_	85.6 nm	(8F) SiO_2_	86.6 nm	(15F) SiO_2_	149.1 nm	(22F) SiO_2_	186.9 nm
Total 2250.0 nm

In the present work, a targeted approach for the design of the multilayered thin films with desired optical properties is presented. Based on computational simulation of the optical properties depending on materials with different refractive indices, thickness, and the number and sequence of layers, multilayer thin films were carefully prepared and thus, efficiently tailoring the optical properties for the possible application for narrow bandpass filters. The introduction of metal films into dielectric-based multilayer thin films open possibilities to efficiently tune the optical properties at specific wavelengths.

## Conclusions

The incorporation of metal thin films in dielectric multilayer thin films is suggested to overcome the low recognition rate of thin film based narrow bandpass filters for the application to biometrics. The results of computational simulations for the desired cutoff frequency of transmittance at specific wavelength bands allowed the effective thickness and number and sequence of layers to be determined. Ti/TiO_2_/SiO_2_ multilayer thin films were deposited using the pulsed-DC reactive sputtering technique, which exhibited a dense structure and smooth surface. The refractive index and extinction coefficient of the Ti, TiO_2_ and SiO_2_ thin films were optimized by controlling the pulsed-DC and RF power during thin film deposition: the refractive indices of Ti, TiO_2_ and SiO_2_ single-layer films at 550 nm was 2.43, 1.48 and 1.99, respectively. Also, the extinction coefficient of the Ti, TiO_2_ and SiO_2_ single-layer thin films is 0, 0, and 3.05, respectively. In comparison to the optical properties of the TiO_2_/SiO_2_ multilayer thin films, the addition of Ti metal thin films (Ti/TiO_2_/SiO_2_) show increased transmittance loss at both low cutoff frequency (480 nm) and high cutoff frequency (400 nm, 680 nm). It is likely that light absorption from metal layer reduces the transmittance at specific wavelength band and thus, effectively enhances the spectral selectivity. It is expected that such a simulation based experimental framework for the design of multilayer thin films will provide an engineering methodology for the development of various application of optical biometrics.

## Methods

### Preparation of thin films

**Figure 9:**
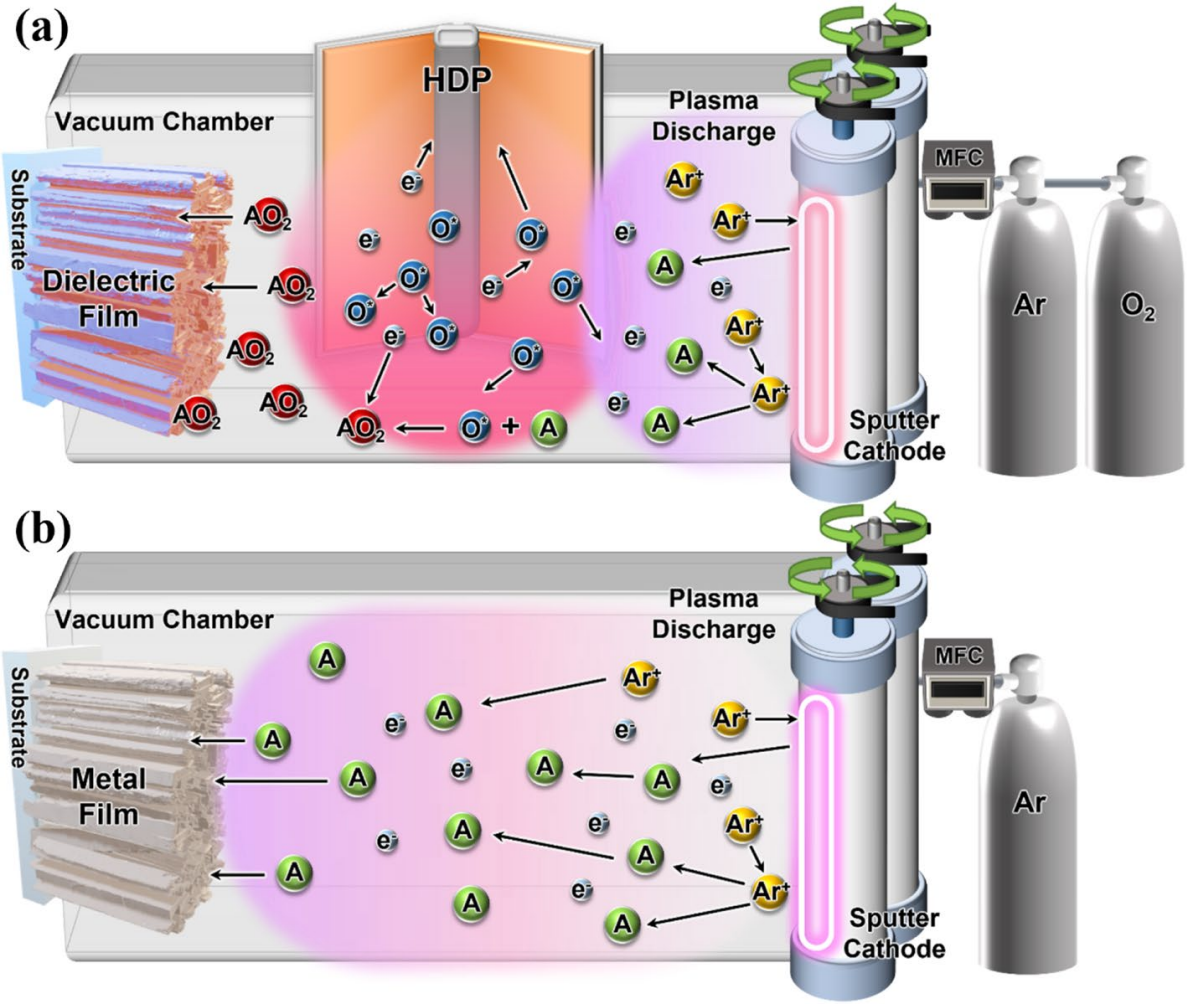
Schematic illustration of pulsed-DC reactive sputtering process of (**a**) dielectric (TiO_2_/SiO_2_) film, and (**b**) metal (Ti) film.

Ti/TiO_2_/SiO_2_ multilayer thin films were deposited on 25 × 25 mm soda-lime glass substrates at room temperature using pulsed-DC reactive sputtering. Initially, the substrates were ultrasonically cleaned for 10 min using isopropyl alcohol, acetone, and distilled water. The Ti and Si targets were cylindrical with a 52 mm diameter and 140 mm length, which is a beneficial size not only to increase target power with efficient cooling but also to decrease the erosion area of the target surface^22^. The distance from the target to the substrate was 150 mm with the target position perpendicular to the substrate, which is an effective configuration for the deposition of dense thin films^23^. The deposition process used to prepare Ti, TiO_2_ and SiO_2_ thin films is illustrated schematically in Fig. 9. Ti thin films were deposited using only the pulsed-DC supply, because for this part of the process, HDP cannot be activated. The TiO_2_ and SiO_2_ thin films were prepared by pulsed-DC power supply with the generation of HDP^38^. Radio Frequency (RF) power was applied using face-to-face electrodes, which increases reactivity and adhesion between substrates and films by accelerating the activation of O_2_ radicals^21,39^. It is noted that when RF power is applied to both the target and HDP, plasma discharge cannot be formed due to interference of RF powers applied on target and HDP, which makes it difficult to form O_2_ radical. Therefore, we applied Pulsed-DC power to the target, which does not induce interference, for deposition of TiO_2_ and SiO_2_ thin films.

Prior to and during thin film deposition, the base pressure of the vacuum chamber was maintained below 1.5 × 10^–5^ torr at 25 °C and relative humidity 25%. The Ti and Si targets were pre-sputtered for 5 min to remove surface impurities. The TiO_2_ and SiO_2_ thin films were prepared by sputtering of targets (Ti, Si) under a mixture of flowing Ar and O_2_ (Ar with 400 standard cubic cm/min and O_2_ with 70 sccm for TiO_2_, and Ar with 250 sccm and O_2_ with 100 sccm for SiO_2_), while Ti thin films were prepared by sputtering of the Ti target under flowing Ar gas with a rate of 250 sccm. The pulsed-DC supply was applied to deposit the TiO_2_, SiO_2_ and Ti thin films with a sputtering power of 13, 8 and 6 kW (and 3 kW), respectively. TiO_2_ and SiO_2_ thin films were deposited under HDP generated by RF sputtering power 1 kW.

### Simulation and design of multilayer thin films

The optimal optical thickness (physical thickness × refractive index) of the multilayer thin films consisting of Ti, TiO_2_ and SiO_2_ was determined using the Essential Macleod Program (EMP, Thin Film Center Essential Macleod v9.6.415)^40,41^. The number of layers, thickness and sequence of layers were automatically calculated to 90% transmittance at 485 nm. Also, Full Width at Half Maximum (FWHM) of the transmittance peak at 485 nm was set to 20 nm for the calculation. It should be noted that transmittance at other spectral wavelengths was set to 0 for the calculation. The refractive index and extinction coefficient of the Ti, TiO_2_ and SiO_2_ in the wavelength range between 300 and 1800 nm for the calculation were obtained using an Ellipsometer (HORIBA Jobin Yvon, UVISEL). The optimal design of the multilayer thin films was determined to be 26 layers with a total thickness of 2.26 µm.

### Materials characterization of thin films

The crystalline and amorphous components of single layers of Ti, TiO_2_ and SiO_2_ films were investigated by X-ray Diffraction (XRD; Malvern Panalytical, Empyrean) in the θ − 2θ mode using monochromatic Cu K_*α*_ radiation. The microstructure of individual Ti, TiO_2_ and SiO_2_ thin films, and Ti/TiO_2_/SiO_2_ multilayer thin films were observed using Field-Emission Scanning Electron Microscopy (FE-SEM; Hitachi, S-4800). Surface roughness of the individual Ti, TiO_2_ and SiO_2_ thin films was measured using Atomic Force Microscopy (AFM; PISA, XE-100), which resulted in a quantitative Root Mean Square (RMS) value for each film. Oxidation states of constituent elements were evaluated by X-ray Photoelectron Spectroscopy (XPS; Ulvac-PHI, PHI 5000 VersaProve). The cross-sectional surfaces of Ti, SiO_2_ and TiO_2_ thin films were examined using several techniques, including High-Resolution Transmission Electron Microscopy (HR-TEM; JEOL, JEM-2100F). Optical transmittance spectra were measured in the wavelength range of 300 nm to 1100 nm using an Ultraviolet–Visible (UV–vis) Spectrophotometer (Agilent, Cary 5000).

## Supplementary Information

Supplementary Figures.
