# Supplementary material for: Ti/TiO2/SiO2 multilayer thin films with enhanced spectral selectivity for optical narrow bandpass filters
Source: Sci Rep. 2022 Jan 7;12:32. doi: 10.1038/s41598-021-03935-z (PMC8741980; doi:10.1038/s41598-021-03935-z)

Supplementary Material

**Ti/TiO_2_/SiO_2_ multilayer thin films with enhanced spectral selectivity for optical narrow bandpass filter**

Dongju Kim^1, +^, Kang Min Kim^2, +^, Hyuksu Han^3^, Junho Lee^1^, Deahyeon Ko^1^, Kyoung Ryeol Park^4^, Kyu-bong Jang^5^, Dongwon Kim^1^, Jennifer Sue Forrester^6^, Seung Hwan Lee^7, *^, Jong Cheol Kim^8, *^, Sungwook Mhin^1, *^

^1^ Department of Advanced Materials Engineering, Kyonggi University, Suwon 16227, Republic of Korea

^2^ Korea Institute of Industrial Technology, 137-41 Gwahakdanji-ro, Gangneung-si, Gangwon 25440, Republic of Korea

^3^ Department of Energy Engineering, Konkuk University, 120 Neungdong-ro, Gwangjin-gu, Seoul 05029, Republic of Korea

^4^ Green Materials & Process R&D Group, Korea Institute of Industrial Technology, 55 Jongga-ro, Jung-gu, Ulsan 44413, Korea

^5^ School of Materials Sicence and Engineering, Inha University, 25 Younghyun-Dong, Incheon 405-751, Republic of Korea

^6^ Analytical Instrument Facility, North Carolina State University, Raleigh, North Carolina 27695, USA

^7^ Department of Mechanical Engineering, Hanyang University, 222, Wangsimni-ro Seongdong-gu, Seoul, 04763, Republic of Korea

^8^ Daegu Mechatronics & Materials Institute, 11 Seongseogongdan-ro, Daegu, 42714, Republic of Korea

^+^These author’s contributed equality to this work.

^*^Correspondence and requests for materials should be addressed to S. H. L (email: [seunghlee@hanyang.ac.kr](mailto:seunghlee@hanyang.ac.kr)) or S. M (email: [swmhin@kgu.ac.kr](mailto:swmhin@kgu.ac.kr)) or J. C. K (email: [jckim@dmi.re.kr](mailto:jckim@dmi.re.kr))

**Figure captions.**

**Figure. S1.** XRD patterns of (a) TiO_2_ thin film, and (b) SiO_2_ thin film.

**Figure. S2.** Process and mechanism for the formation of dense thin films by Pulsed-DC reactive sputtering: (a) Ti thin film, (b) TiO_2_ thin film, (c) SiO_2_ thin film.

**Figure. S3.** Refractive Index (n) and Extinction Coefficient (k) as a function of sputtering parameters: (a) Ti thin film, (b) TiO_2_ thin film, (c) SiO_2_ thin film.

**Figure. S4.** Morphology and transmittance spectra of multi-layered structures of Ti/TiO_2_/SiO_2_ films with 7-8 layers: (a) Cross-sectional SEM micrograph of multilayer film with Ti 4 nm (b) Cross-sectional SEM micrograph of multilayer film with Ti 8 nm, and (c) Cross-sectional SEM micrograph of multilayer film with Ti 11 nm.

**Figure. S5.** Morphology and layer thickness multi-layered structure of TiO_2_/SiO_2_ films with 23 layers.

**Figure. S6.** Simulated transmittance spectra of multilayer films of (a) Si/TiO_2_/SiO_2_ films with 26-layers, (b) Ag/TiO_2_/SiO_2_ films with 26-layers, (c) Zn/TiO_2_/SiO_2_ films with 26-layers, and (d) Al/TiO_2_/SiO_2_ films with 26-layers.

**Figure. S7.** Transmittance spectra of multilayer films of (a) Ti/TiO_2_/SiO_2_ films with 26-layers, (b) 27-layers. (c) 28-layers, and (d) 29-layers. Simulated transmittance spectra are highlighted by the dotted line.

**Figure. S1.**


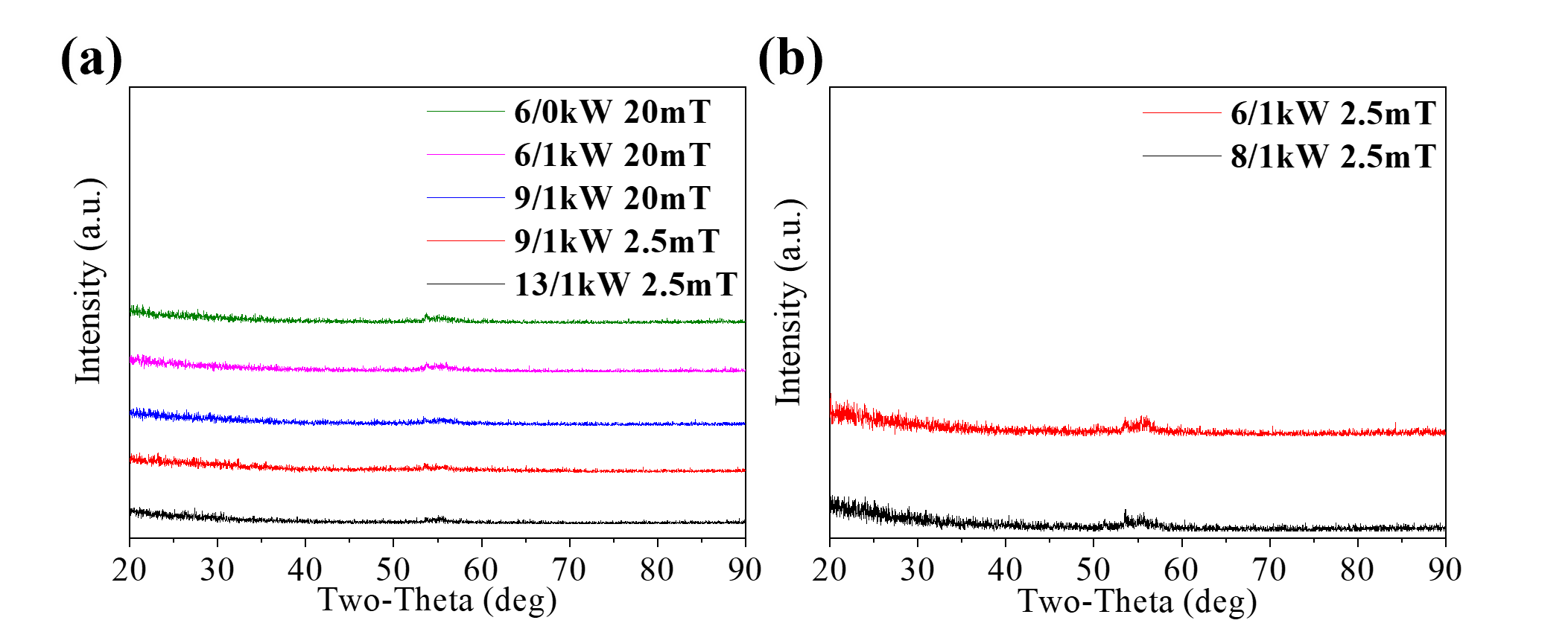


**Figure. S2.**


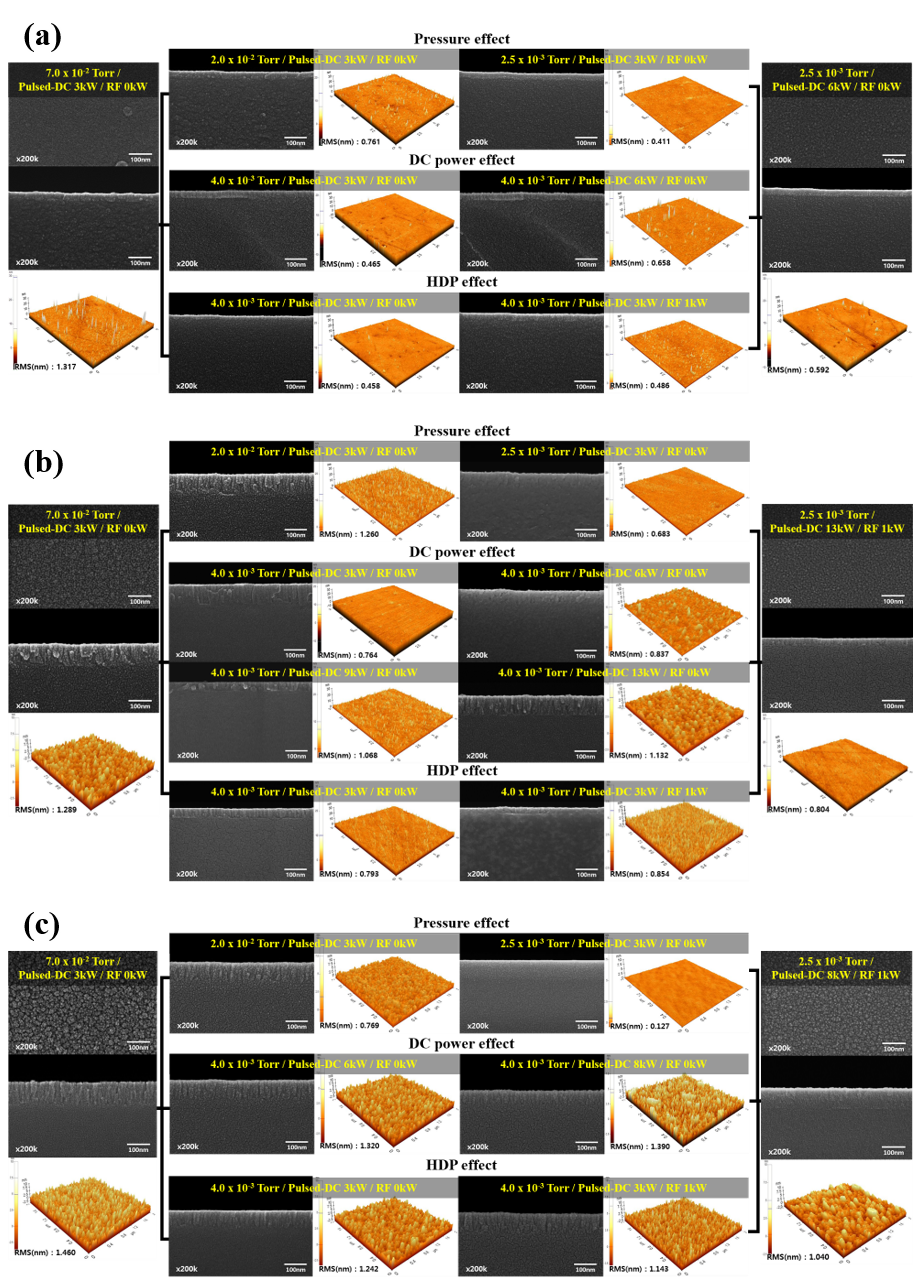


**Figure. S3.**


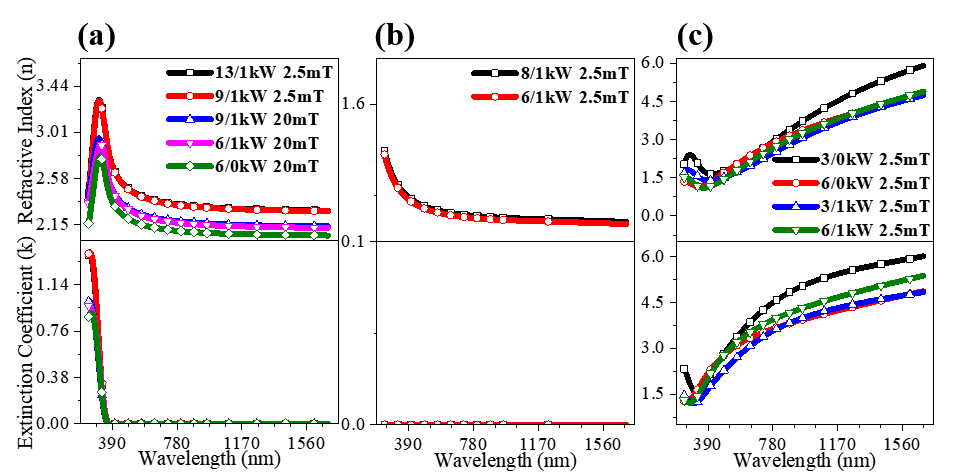


**Figure. S4.**


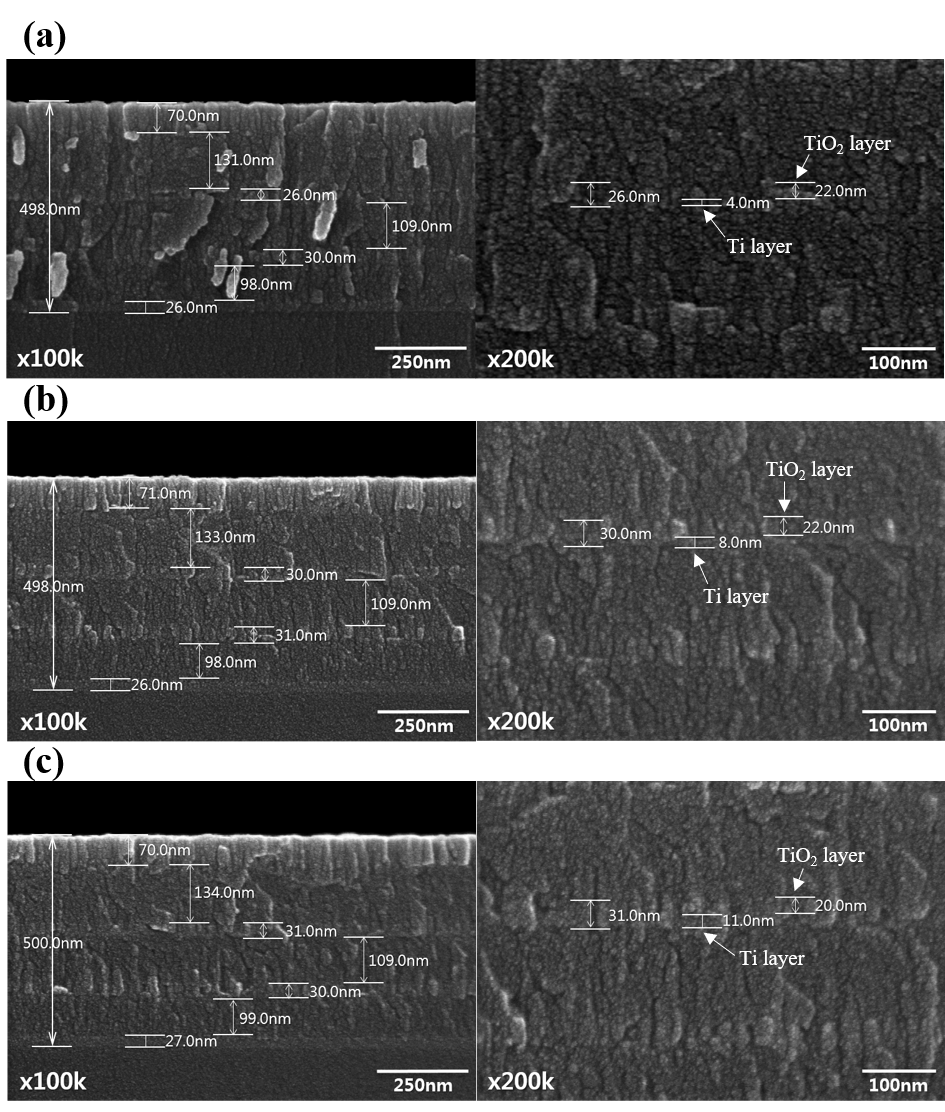


**Figure. S5.**


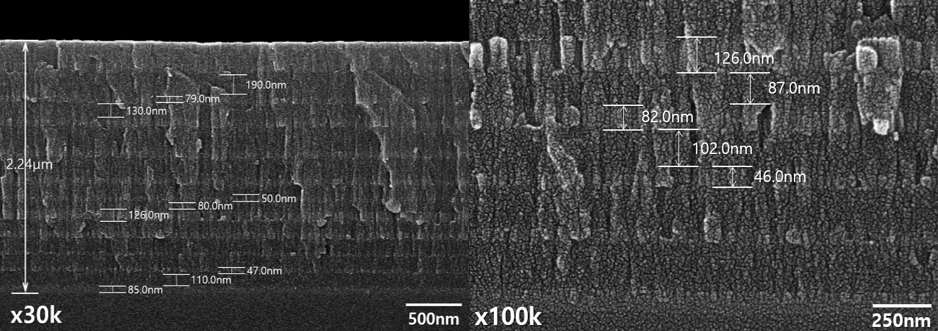


**Figure. S6.**


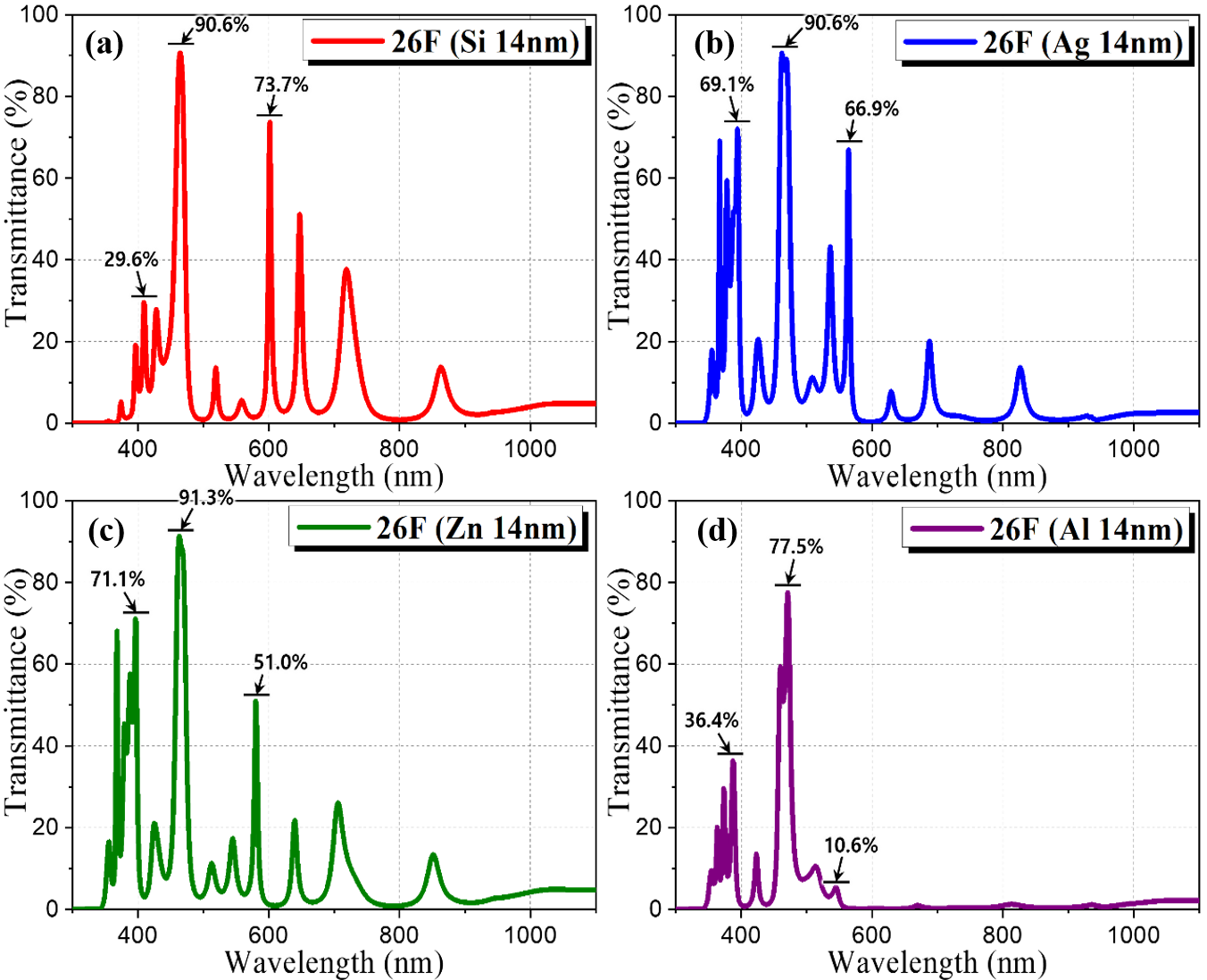


**Figure. S7.**


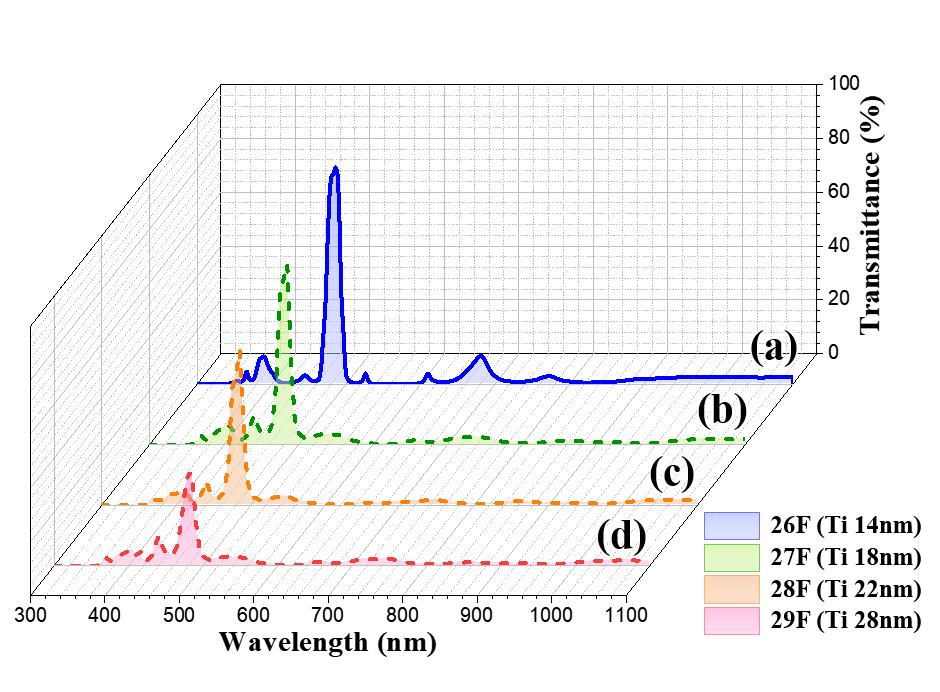

Supplement: Supplementary file 1 — Supplementary Figures. [file 41598_2021_3935_MOESM1_ESM.docx]
